# Roles of Adiponectin Signaling Related Proteins in Mammary Tumor Development

**DOI:** 10.14744/less.2019.85688

**Published:** 2019-12

**Authors:** Bilge Güvenç Tuna, Margot Cleary, Soner Dogan

**Affiliations:** 1 Department of Biophysics, Yeditepe University Faculty of Medicine, İstanbul, Turkey; 2 University of Minnesota, Hormel Institute Medical Research Center, Austin, MN, USA; 3 Department of Medical Biology, Yeditepe University Faculty of Medicine, İstanbul, Turkey

**Keywords:** Adiponectin, adiponectin receptor, liver, mammary fat pad, mammary tumor, Adiponektin, adiponektin reseptör, karaciğer, meme dokusu, meme tümörü

## Abstract

**Objective:**

This study aims to investigate the expression levels of adiponectin signaling related proteins in mammary tissue, liver and breast cancer tissue in mice. Adiponectin, an adipocytokine, is secreted from adipose tissue and has been documented to have roles in diabetes, inflammation, and cancer development. In particular, levels of serum adiponectin are inversely associated with obesity and a decrease in serum adiponectin levels have been reported to be associated with breast cancer. There are two adiponectin receptor subtypes, AdipoR1 and AdipoR2, which have been identified in mammalian tissues, including human cancer cell lines and also in human mammary tumors. However, the role of adiponectin receptors in breast cancer development remains to be established.

**Methods:**

In this study, MMTV-TGF-α transgenic mice were fed from week 10 up to week 74 of age. Expression levels of adiponectin, AdipoR1 and AdipoR2 proteins were measured in the mammary fat pad (MFP), mammary tumor (MT) and liver tissues from 74 weeks old MMTV-TGF-α transgenic mice with and without MT using Western Blot. Adiponectin levels were measured using ELISA assay.

**Results:**

Protein expression levels of Adiponectin and AdipoR1 were significantly lower in MTs compared to control tissues. However, AdipoR2 protein expression levels were similar in MT and MFP tissues from MT-positive and MT-negative mice. The expression levels of adiponectin, AdipoR1 and AdipoR2 proteins in liver tissues were also similar in MT-positive and MT-negative mice. Serum adiponectin levels of the MT-positive and MT-negative mice were similar.

**Conclusion:**

These results indicate that adiponectin and its receptors are differentially regulated depending upon the specific tissue analyzed. AdipoR1 and adiponectin may play important roles in MT development.

## Abstract (tr)

**Amaç:**

Bu çalışmanın amacı fare karaciğer, meme dokusu ve meme tümörü dokularındaki adiponektin sinyal yolağı ile ilişkili proteinlerin ekspresyon seviyelerinin belirlenmesidir. Adiponektin reseptörünün, AdipoR1 ve AdipoR2 olmak üzere memeli dokusunda belirlenmiş iki alt tipi vardır. Serum adiponektin seviyelerinin meme kanseri ile ilişkili olduğu bildirilmiştir. Fakat, adiponektin reseptörlerinin meme tümörü oluşumundaki rolü tam olarak ortaya konmamıştır.

**Gereç ve Yöntem:**

MMTV-TGF-α transgenik fareler 10 haftalıktan 74 haftalığa kadar beslendi. Meme tümörü geliştiren ve geliştirmeyen 74 haftalık transgenik farelerin karaciğer, meme (MFP) ve meme tümörü (MT) dokularında adiponektin, AdipoR1 ve AdipoR2 proteinlerinin ekspresyon seviyeleri western blot yöntemi kullanılarak belirlendi. Adiponektin seviyesi ELISA yöntemi ile ölçüldü.

**Bulgular:**

Adiponektin ve AdipoR1 protein ekspresyon seviyeleri MT geliştiren farelerde, MT geliştirmeyen farelere göre anlamlı olarak daha azdı. Fakat, MT-pozitif ve MT-negatif farelerin MT ve MFP dokularındaki AdipoR2 proteininin ekspresyon seviyeleri benzerdi. MT-pozitif ve MT-negatif farelerin karaciğer dokusundaki adiponectin, AdipoR1 ve AdipoR2 protein ekspresyon seviyeleri de benzerdi. Ek olarak, MT-pozitif ve MT-negatif farelerin serum adiponectin seviyeleri benzerdi.

**Sonuç:**

Bu sonuçlar adiponektin ve reseptörlerinin analiz edilen dokuya spesifik bağımlı olarak düzenlendiğini işaret etmektedir. Ayrıca, AdipoR1 ve adiponectin MT gelişiminde önemli rol oynuyor olabilir.

## INTRODUCTION

Adiponectin, an adipocytokine, has been proposed to play crucial roles in different physiological and pathophysiological events, such as regulation of lipid and glucose metabolism. Specifically, it plays anti-atherogenic, anti-diabetic, anti-inflammatory, neuroprotective, and anti-tumorogenic roles.^[1–6]^ A recent study showed that both adiponectin receptors are involved in regulating cell membrane fluidity in cancer cells.^[7]^

There are two main adiponectin receptors, adiponectin receptor-1 (AdipoR1) and adiponectin receptor-2 (AdipoR2), that have been identified and their expression has been reported in numerous tissues, such as the endometrium, brain and breast tissues obtained from both in humans and animals.^[3,6–11]^ On the other hand, both of adiponectin receptors’ expression has also been reported in cancer cell lines and tissues, including breast cancer and endometrial cancer, in which expression of AdipoR1 was shown to be higher compared to that of AdipoR2.^[9]^ In addition to in-vivo studies, the presence of adiponectin and its receptors have also been shown in in-vitro studies as well.^[7–9,12–14]^ For example, adiponectin receptors mRNA expression levels were shown in the human mammary tumor, pancreatic, lung and endometrial cell lines.^[7–9,11–14]^ It was reported that adiponectin functions through its receptors via modification of inflammatory signaling molecules, including Erk1/2, Akt, TNFα, IL-1β, NFkB, IL-6, IL-8 and MCP1.^[14]^ Another main target for adiponectin protein is liver tissue where it is survival and apoptotic effects were observed in hepatic cells.^[15]^ All these findings have implied the possible involvement of adiponectin and its associated signaling proteins in anti-cancer development activity.

Animal models are useful for mechanistic, prospective and intervention studies to investigate molecular aspects of cancer development and prevention. In this study, we utilized an established mammary tumor model (MMTV-TGF-α mice) that develops hormonally responsive tumors, mostly later stages of their life. Thus, the present study aims to show whether adiponectin and/or its receptors were differentially expressed in mammary, tumor and liver tissues of MT bearing animals compared to the tumor-free tissues. In addition, we reported protein expression levels of AdipoR1, AdipoR2 and adiponectin in the mammary tumor (MT), mammary fat pad (MFP) and liver tissues in relationship to serum adiponectin levels in this breast cancer mouse model.

## MATERIALS AND METHODS

### Study design

All procedures with live animals were performed according to the guidelines and approval of the University of Minnesota Institutional Animal Care and Use Committee in an AAALAC accredited facility. Mice used in this study were MMTV-TGF-α transgenic mouse models, which were developed in Dr. Coffey’s laboratory.^[16]^ These mice over-express a growth factor called; human TGF-α and around 60% of them develop MT at the postmenopausal stage. The mice were obtained using the breeding protocol and genotyping assay previously described and fed the AIN-93M diet (Harlan Teklad, Madison, WI) from eight until 74 weeks of age.^[17]^ Over the course of the experiment, serum samples were obtained via orbital bleeding at 10, 19, 25, 28, 40, 49 and 74 weeks of age. Then, mice were sacrificed, dissected and liver tissues, MFP, or suspected MT were collected. A portion of the samples was sent in formalin (10%) to the Department of Pathology for histopathological analyses in a blinded fashion. All confirmed MT samples were histopathologically grade two. The rest of the tissue samples were stored at −80°C for future analyses. According to MT status, animals were assigned into two groups; those with tumors, MT-positive (n=8), and those without tumors, MT-negative (n=8). If the mouse was designated as MT-positive, its suspected MT tissue was termed an MT, but if the mouse was MT-negative, the tissue was called control.

### Western blot analysis for adiponectin and its receptors

Individual animal samples were minced in the extraction solution with protease inhibitors (G-Biosciences/Genentech, St. Louis, MO). Tissue Total Protein Extraction (TPER) reagent was used for total protein extraction according to manufacturer’s protocol (Pierce Corp, Rockford, IL) and measured using the Bio-Rad protein assay kit with BSA as a standard (Bio-Rad, Hercules, CA). Then, total proteins were electrophoresed on 4–15% of polyacrylamide gradient gels and transferred to a polyvinylidene difluoride (PVDF) membrane (Immobilon-P, Millipore, Billerica, MA). Tris-Base solution with 1% milk concentrate and 0.1% Tween-20 was used for blocking blots. The membranes were incubated with appropriate primary antibodies against adiponectin itself (ProSci Inc., Poway, CA), AdipoR1 (Santa Cruz), AdipoR2 (Santa Cruz) and β-actin (Delta Biolabs, Vandell Way Campbell, CA) proteins in tissue samples. Consequently, membranes were incubated with alkaline phosphatase-conjugated goat anti-mouse IgG as a secondary antibody (Santa Cruz). Enhanced Chemifluorescence (ECF substrate) was obtained from Amersham Biosciences (Piscataway, NJ) to visualize the bands using a Storm 840 Imaging System (Amersham Biosciences, NJ). To compare molecular weights (MW) of the visualized proteins, standard MW markers were run simultaneously. To quantify the intensity of WB bands, the program called the UN-SCAN-IT gel densitometry analysis method was used (Silk Scientific, Orem, UT). All measurements were normalized with β-actin from the same sample. For WB analysis, eight different animal samples per group were used.

### Measurement of serum adiponectin levels

Blood collection was performed five hours after animals were given the amount of their daily food at all time points. To determine if changes in serum adiponectin preceded MT development, serum samples were obtained at multiple age points (10, 19, 25, 28, 40, 49 and 74 weeks old). Adiponectin levels in serum were measured with a commercial RIA kit per manufacturer’s protocol (LINCO Research, MO).

### Statistical analysis

The data were presented as means ± SEM. Statistical significance was tested by using GraphPad Prism 7.0 and determined by Student’s t-test. When P-value is <0.05, statistical significance was indicated by *. The number of the individual animals was eight unless it is indicated in each figure legends. It was indicated with “n” in each group.

## RESULTS

### Body Weights, Mammary Fat Pad Weights and Mammary Tumor Detection:

The final body weights of the MT-positive mice were calculated by subtracting MT weights from the total body weight values. However, no significant differences were detected between MT-positive and MT-negative mice concerning BWs, parametrial, MFP, retroperitoneal, and total fat pad weights, as shown in Table 1. None of MTs were detected by palpation in these animals before the terminal age, 74 weeks. The average weight of the MTs in the MT-positive mice was approximately half a gram (Table 1).

### Adiponectin Receptor-1 (AdipoR1) and Adiponectin Receptor-2 (AdipoR2) Expression Levels in MT and MFP:

**Figure 1.**
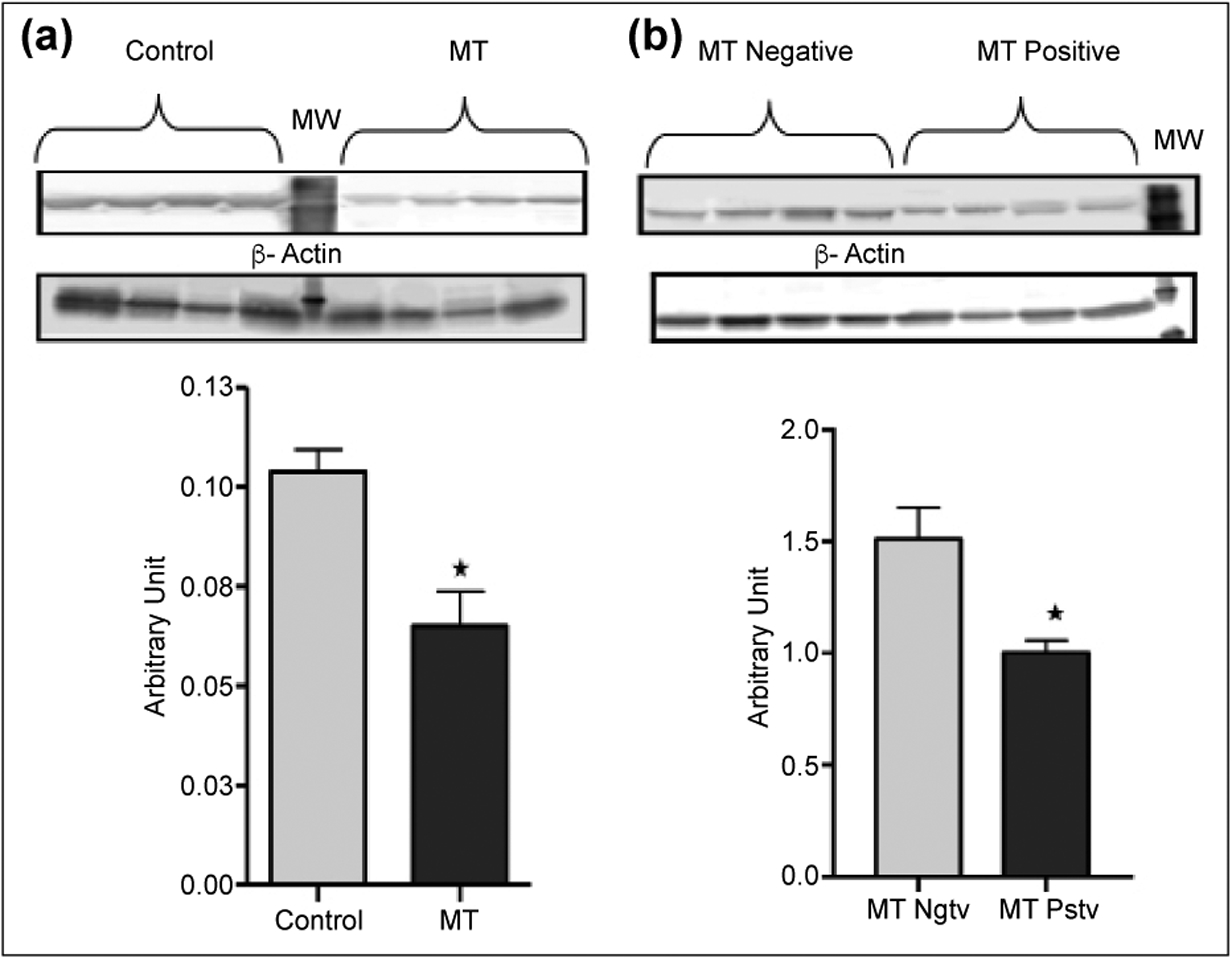
Protein expression levels of AdipoR1 in animals with and without MT after 74 weeks of age. AdipoR1 protein expression levels in MT or control tissues (Panel A) and in MFP tissues (Panel B) were measured using western blot analysis as explained in the materials and methods section. Control tissues were removed from the same location where MT grows in MT-positive animals at the same age. Data shown here are the average density values of eight individual mice in each group (n=8). MFP and either MT or control tissues were taken from the same animal for each “n” values. *Indicates statistically significant difference (p=0.0022 for panel A, and p=0.0073 for panel B). MW stands for molecular weight marker. MT: Mammary tumor; MW: Molecular weights.

**Figure 2.**
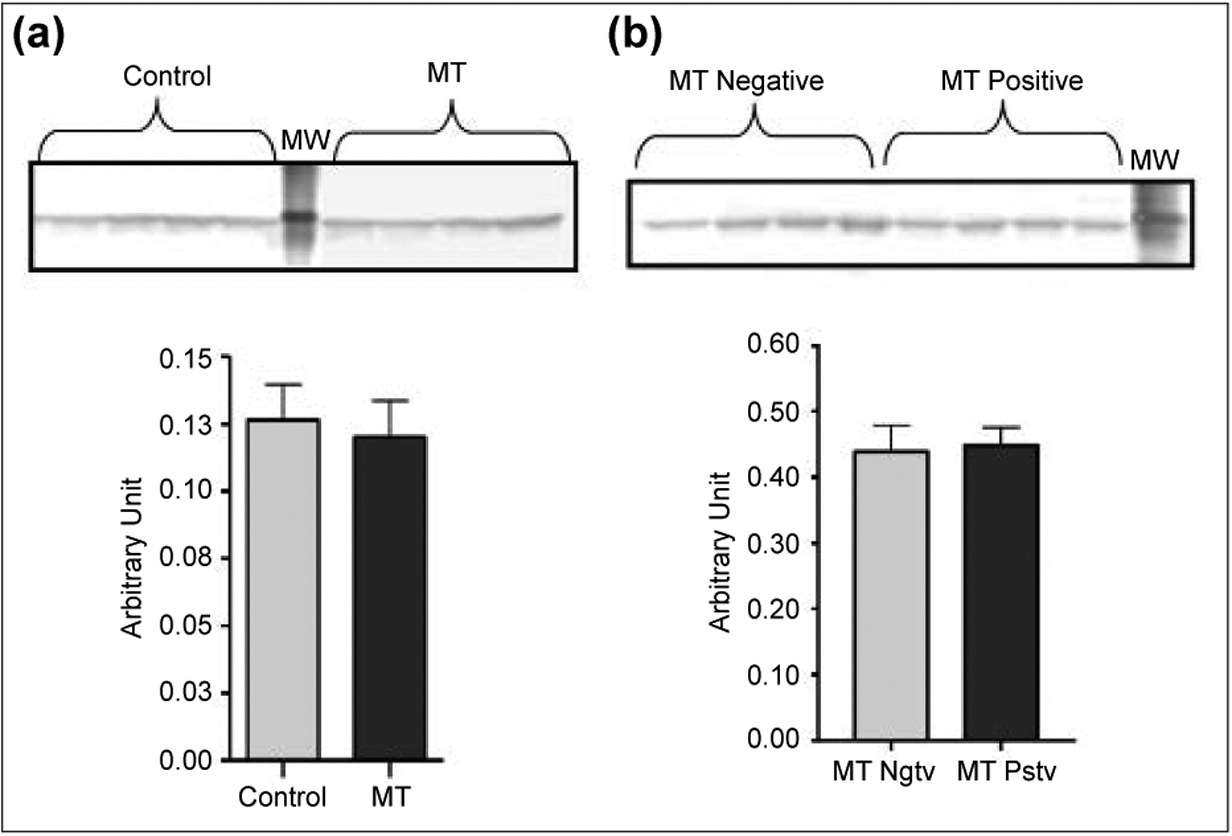
Protein expression levels of AdipoR2 in animals with and without MT after 74 weeks of age. AdipoR2 protein expression levels in MT or control tissues (Panel A) and in MFP tissues (Panel B) were measured using western blot analysis as explained in the materials and methods section. Control tissues were removed from the same location where MT grows in MT-positive animals at the same age. Data shown here are the average density values of eight individual mice in each group (n=8). MFP and either MT or control tissues were taken from the same animal for each “n” values. MW stands for molecular weight marker. MT: Mammary tumor; MW: Molecular weights.

MT tissue from MT-positive animals had significantly lower AdipoR1 protein expression levels compared to control, non-tumor, tissue obtained from the same location in MT-negative mice (Fig.1a) (p=0.002). Expression levels of AdipoR1 proteins in MFP tissue from the MT-positive mice were also significantly lower than levels obtained from MFP tissue from MT-negative mice (Fig. 1b) (p=0.007). However, AdipoR2 protein levels were not significant neither in MT nor MFP tissues of the MT-positive or MT-negative mice (Fig. 2) (p>0.05).

### Adiponectin Expression Levels in MT and MFP:

**Figure 3.**
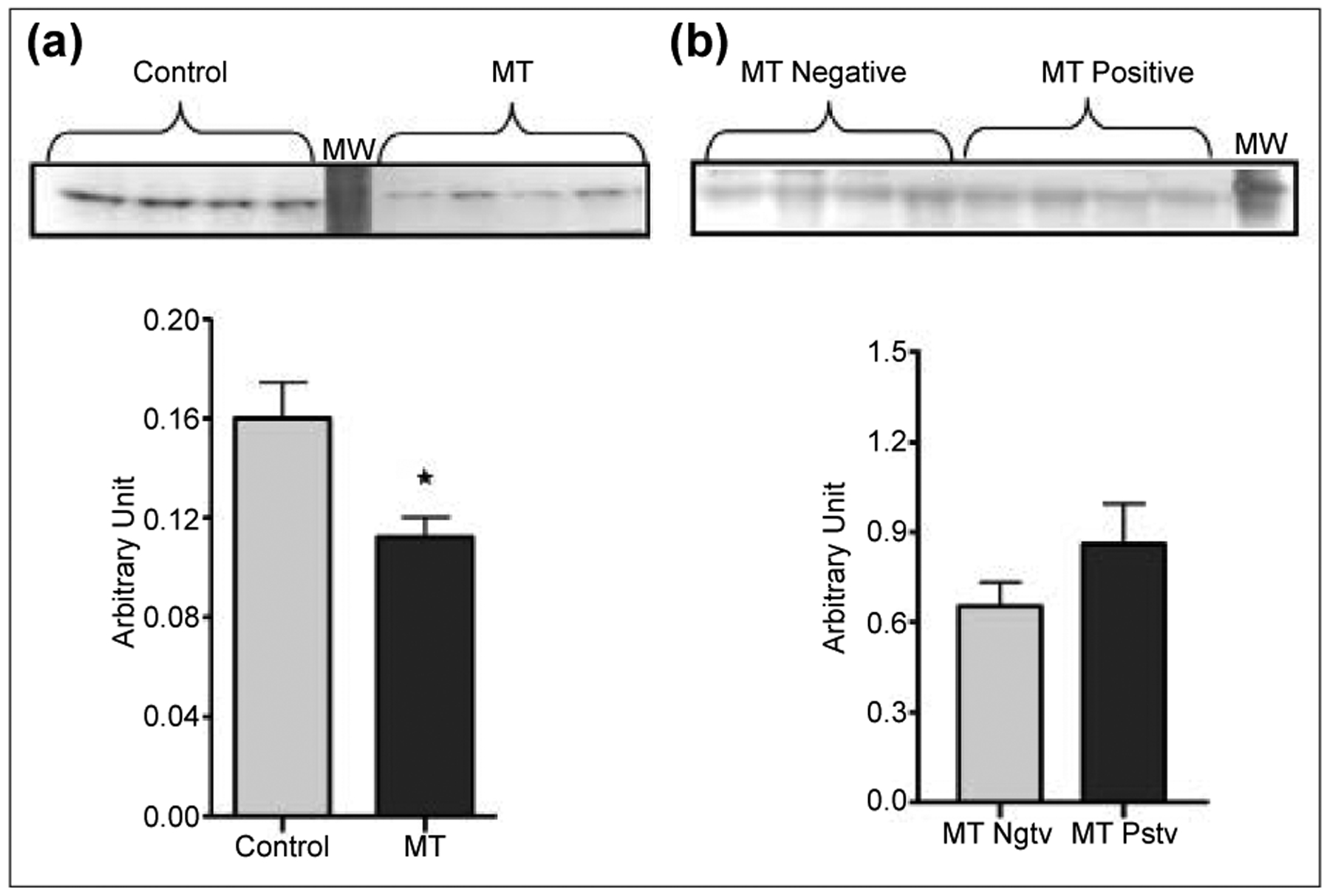
Protein expression levels of adiponectin in animals with and without MT after 74 weeks of age. Adiponectin protein expression levels in MT or control tissues (Panel A) and in MFP tissues (Panel B) were measured using western blot analysis as explained in the materials and methods section. Control tissues were removed from the same location where MT grows in MT-positive animals at the same age. Data shown here are the average density values of eight individual mice in each group (n=8). MFP and either MT or control tissues were taken from the same animal for each “n” values. *Indicates a statistically significant difference (p=0.015). MW stands for molecular weight marker. MT: Mammary tumor; MW: Molecular weights.

Adiponectin protein expression level was significantly lower (p=0.015) in MT samples from the MT–positive mice compared to the adiponectin levels in control tissue from the MT-negative mice (Fig. 3a). However, there was no significant difference (p>0.05) for adiponectin protein expression levels in MFP samples from the two groups (Fig. 3b).

### Adiponectin and its Receptor Expression Levels in Liver:

**Figure 4.**
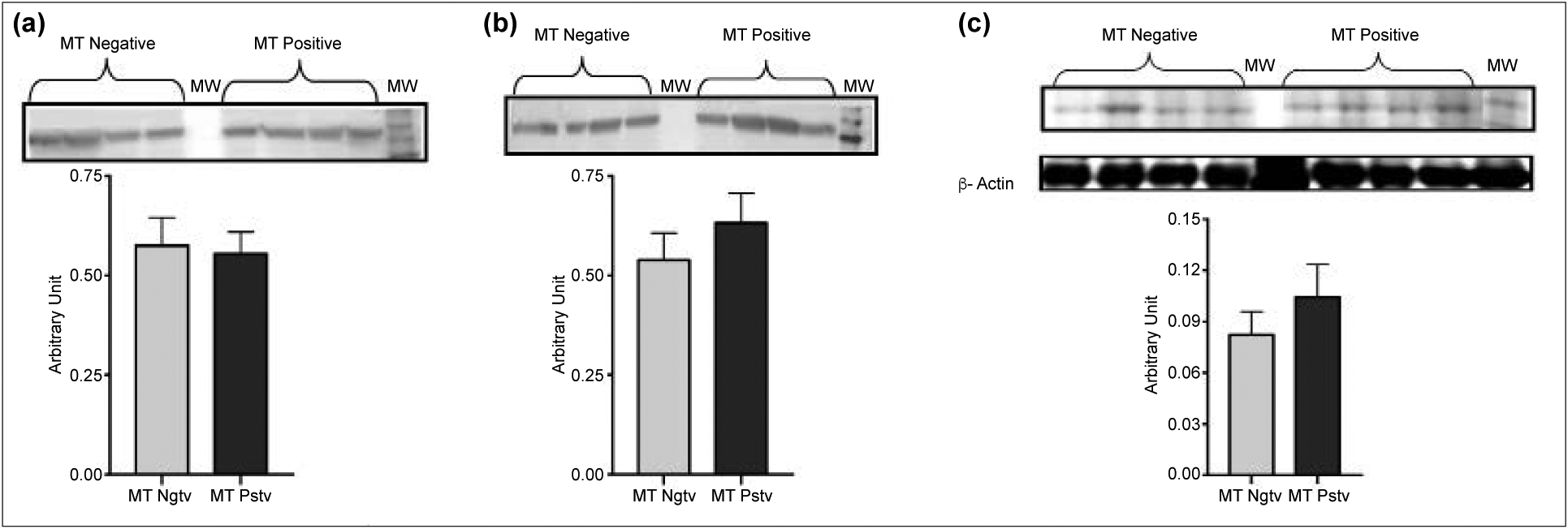
Protein expression levels of AdipoR1, AdipoR2 and Adiponectin in liver tissues of the animals with and without MT after 74 weeks of age. AdipoR1 (Panel A), AdipoR2 (Panel B) and Adiponectin (Panel C) protein expression levels in liver tissues were measured using western blot analysis as explained in the materials and methods section. Data shown here are the average density values of eight individual mice in each group (n=8). MW stands for molecular weight marker. MT: Mammary tumor; MW: Molecular weights.

There were no statistically significant (p>0.05) differences for AdpoR1 (Fig. 4a), AdipoR2 (Fig. 4b), and adiponectin (Fig 4c) protein expression levels in the liver from the MT-positive animals compared to the MT-negative animals (Fig. 4).

### Serum Adiponectin Levels:

**Figure 5.**
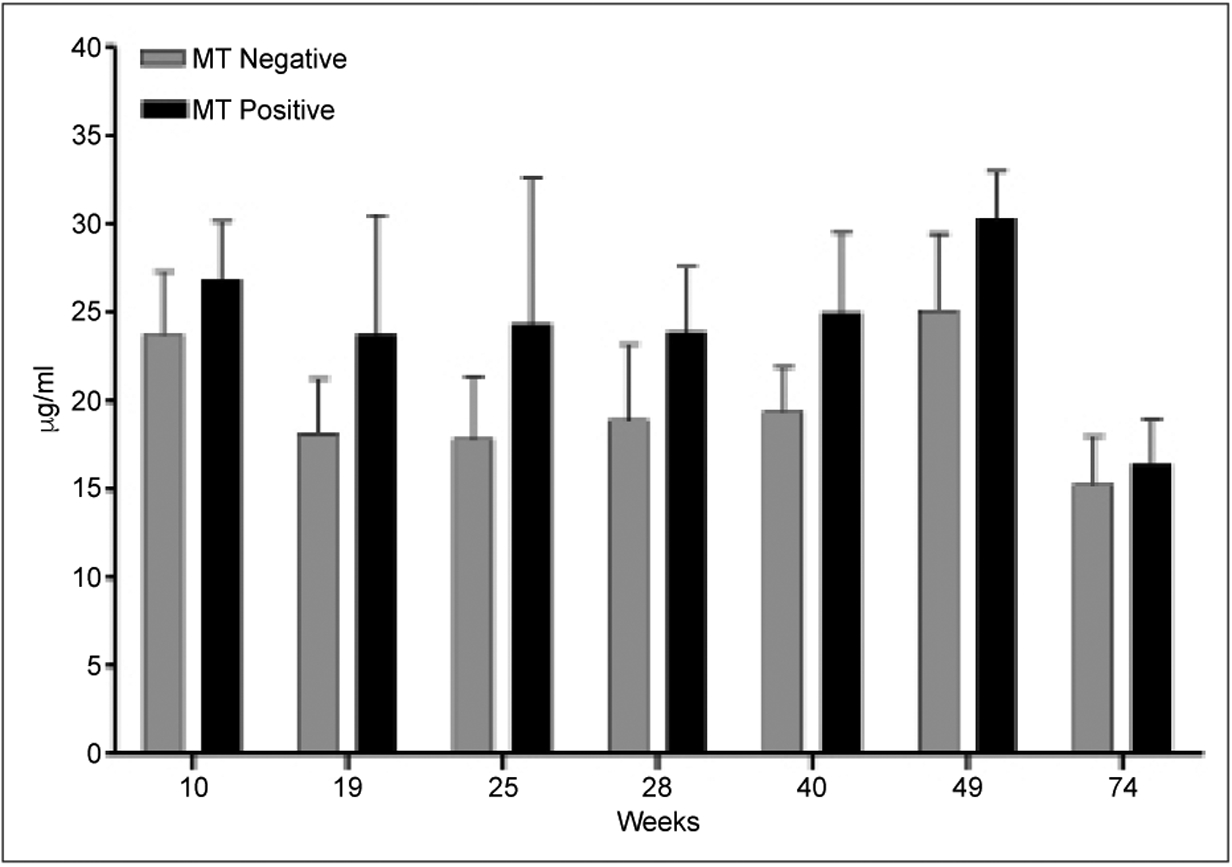
Serum adiponectin levels of the animals with and without MT. The serum samples were collected at different time points, starting from week 10 up to week 74. Serum adiponectin levels were measured as explained in the materials and methods section. Although serum adiponectin level is an average of seven to eight individual mice for week 74 (n=7–8), it is three to seven (n=3–7) for earlier time points. MT: Mammary tumor.

Levels of serum adiponectin of MT-positive and MT-negative mice were similar at 74 weeks of age (Fig. 5). The average serum adiponectin for MT-positive mice was 15.58±1.68 μg/ml and 15.24±2.75 μg/ml, for the MT-negative mice (p>0.05). There were also no significant (p>0.05) differences between the two groups at earlier time points, 10, 19, 25, 28, 40 and 49 weeks.

## DISCUSSION

In the present study, we found that AdipoR1 and adiponectin protein expression levels were significantly lower in MT tissues compared to control tissues obtained from MMTVTGF-α mice at 74 weeks. Although AdipoR1 protein expression level was significantly lower in MFP tissues of the MT-positive mice compared to MT-negative mice, the expression level of AdipoR2 and adiponectin protein in MFP was similar in both groups. Furthermore, there were no significant differences for adiponectin, AdipoR1 and AdipoR2 protein expression levels in liver tissue samples from mice with and without MT. These results indicate that AdipoR1 and AdipoR2 receptors are differentially expressed at the tissue level. These data also suggest that AdipoR1 and adiponectin protein expression may play important roles in adiponectin signaling pathways during MT development since their levels were significantly different between the MT-positive and MT-negative mice. Our results coincide with the previous findings, which also report that AdipoR1 is elevated in cancer development but not AdipoR2.^[9,10,11]^ In this context, Rogozina et al.^[10]^ have reported changes in adiponectin and its receptor expression levels either in mammary tissue or MT tissue obtained from a group of mice subjected to different calorie restriction protocols. They found lower expression level of AdipoR1 protein in MFP of mice with the higher MT incidence rate (either ad-libitum or chronic calorie-restricted groups) compared to intermittent calorie-restricted group of mice, which had the lower MT incidence rate.^[10]^ In the same study, no significant difference was reported for expression levels of either adiponectin or AdipoR2 proteins. In addition, Rogozina et al.^[10]^ also documented that expression levels of adiponectin protein in MFP tissues from most of the tumor-bearing mice were undetectable. Moreover, they reported significantly lower AdipoR2 protein expression levels in MT samples obtained from intermittently restricted mice compared with either ad-libitum or chronic calorie-restricted groups. Their results support the hypothesis of the current paper that adiponectin signaling related proteins may play important protective roles at the local tissue level and AdipoR1 may be more important than AdipoR2 through adiponectin signaling in MT development. It is also worth mentioning that Rogozina et al.^[10]^ did not compare the tissue samples from mice developed MT with that of the samples from MT free mice in the same experiment. They just reported calorie restriction effects on the adiponectin signaling related proteins expression levels.

Our results, in general, are compatible with the findings of the previous study, which used human tissue samples. They indicated that the mRNA expression level of adiponectin was significantly higher in the healthy mammary tissue from subjects without cancer compared to MT samples from women diagnosed with cancer.^[12]^ The level of AdipoR1 mRNA expression in healthy mammary tissue, which was adjacent to the MT, was also higher than that of in the MT itself. However, mRNA expression levels of AdopoR1 in the MT samples were higher compared to the control/healthy mammary tissue samples from the healthy subjects. [^12^] In the same study, there were no significant differences for mRNA expression levels of AdipoR2 among control tissue, MT and adjacent healthy mammary tissues.^[12]^ Higher adiponectin levels in breast tumors could be explained by the anti-inflammatory or protective effects of adiponectin against tumor development. It is likely the body secretes more adiponectin to guard itself against cancer development. Takahata et al.^[18]^ suggested that AdipoR1 plays a major role in adiponectin signaling in the mammary tissue because they reported AdipoR1 but not AdipoR2 expression in breast stromal cells. These earlier observations support the findings in the present study. We have also measured lower adiponectin protein expression levels in MT tissue samples compared to the control tissue samples. On the other hand, compared to healthy control mammary tissue samples, significantly higher adiponectin level in mammary tissue of breast cancer patients has been reported.^[19]^ In the same study, there was no correlation between tissue adiponectin levels and either mammary tumor stage or tumor size. The major difference between the present study and the previous study is although, in the previous study, tissue adiponectin levels were measured by ELISA and reported, serum adiponectin levels were not reported.^[19]^ In the present study, both serum adiponectin levels and tissue protein expression levels were measured and reported. Also, in this study, we report the differential expression of adiponectin receptors subtypes, AdipoR1 and AdipoR2 in different tissues from mice with or without MTs although there is no significant difference in serum adiponectin levels in these mice. Our results coincide with the previous findings since the differential expression of two different adiponectin receptor subtypes under different physiological conditions were reported at the tissue levels while no significant difference in the serum adiponectin levels was measured in other studies.^[20]^ In another study, peptide-based adiponectin receptor agonist drug treatment of cancer cells also supported our hypothesis of the involvement of adiponectin receptors in cancer development, i.e., a peptide that mimics adiponectin showed promising results inhibiting cancer cell proliferation by 31% in xenografts study.^[21]^

Previously, although some studies reported a negative correlation between serum adiponectin levels and postmenopausal MT development,^[12,22,23]^ other studies did not find a significant difference in serum adiponectin levels in association with postmenopausal breast cancer or MT development.^[5,22,23]^ In general, higher serum adiponectin levels have been correlated with reduced breast cancer risk (up to 65%). In the present study, we measured serum adiponectin levels before and at the time MTs were detected at necropsy, but no significant differences were found (p>0.05) between the two groups. However, the MT-positive group always had a slightly higher serum adiponectin level. This may be a consequence of the relatively small number of mice used in comparison to human epidemiological studies. It should be mentioned that there are some previous studies that also found no significant difference between MT developed group and the control ones. Our findings of no difference in serum adiponectin levels between MT developed and MT free groups are also similar to previous findings.^[24]^

In conclusion, adiponectin and AdipoR1 protein expression levels were significantly lower in MT compared to control tissues in MMTV-TGFα mice. In addition, protein expression level AdipoR1 was significantly lower in the MFP of mice with MTs at 74 weeks of age. However, AdipoR2 protein expression level was similar in MT and MFP tissues in MT-positive and MT-negative mice. Additionally, levels of AdipoR1, AdipoR2 and adiponectin itself protein expression in the liver were not influenced by the presence of MTs. The current results demonstrate that two adiponectin receptors are differentially regulated in different tissues. Adiponectin itself and AdipoR1 at the mammary tissue level may play important roles in the development of breast cancer. Mammary tumorigenesis in MMTV-TGF-α transgenic mouse model has been shown to be responsive to circulating leptin levels^[25]^ and to dietary interventions.^[17]^ Thus, it is anticipated that the role of elevated adiponectin in protection from mammary tumorigenesis or the role of reduced adiponectin in obesity in enhancing MT development can now be investigated in detail using the mouse model in the current study since cancer development in human is more complex.^[26–28]^ Although these findings lead us to conclude that AdipoR1, rather than AdipoR2, might be dominant in adiponectin signaling in MT development, further studies are necessary to characterize the roles of these two receptors in tumor development.

## Figures and Tables

**Table 1.** Average of final body weight, MT, parametrical, retroperitoneal, total mammary fat pad weights of MT-positive and MT-negative MMTV-TGF-α mice. Final body weight of MT-positive mice was calculated by subtracting MT weights from total body weights

Weights in grams	MT-negative	MT-positive	p
Body weight	34.95±2.80	35.87±2.07	0.795
Mammary tumor	NA	0.516±0.11	NA
Parametrial fat pad	1.91±0.55	1.91±0.40	0.999
Retroperitoneal fat pad	0.43±0.12	0.41±0.06	0.917
Total mammary fat pad	0.96±0.13	0.90±0.11	0.717
Total fat pad	4.26±0.99	4.11±0.75	0.910

MT: Mammary tumor; MMTV: Mammary tumor model.
